# Oxymatrine ameliorates *Malassezia* overgrowth-induced psoriasis *in vivo* and *in vitro* by inhibiting the biofilm formation and inflammation

**DOI:** 10.1080/21501203.2025.2511903

**Published:** 2025-06-10

**Authors:** Miao-Miao Liu, Jie Bai, Zi-Ye Tian, Ting-Ting Zheng, Teun Boekhout, Qi-Ming Wang

**Affiliations:** a School of Life Sciences, Institute of Life Sciences and Green Development, Hebei University, Baoding, China; b College of Sciences, King Saud University, Riyadh, Saudi Arabia; c The Yeasts Foundation, Amsterdam, the Netherlands; d Hebei Basic Science Center for Biotic Interaction, Hebei University, Baoding, China; e Engineering Research Center of Ecological Safety and Conservation in Beijing-Tianjin-Hebei (Xiong’an New Area) of MOE, Hebei University, Baoding, China

**Keywords:** Oxymatrine, *Malassezia*, psoriasis, biofilm, inflammation

## Abstract

The basidiomycetous yeast genus *Malassezia* is involved in the exacerbation of psoriatic lesions. Oxymatrine (OMT), a quinoline alkaloid derived from *Sophora flavescens*, exhibits diverse pharmacological properties, including anti-inflammatory, anticancer, and antiviral effects. However, whether OMT exerts therapeutic effects against *Malassezia*-associated psoriasis remains unclear. This work aimed to study the antifungal and antibiofilm effect of OMT on several *Malassezia* species and the therapeutic benefits of OMT on *Malassezia*-associated psoriasis *in vivo* and *in vitro*. Treatment with 0.64 mg/mL OMT showed decreasing levels of biofilm formation of *Malassezia* species. Histomorphology and functional analyses demonstrated that OMT treatment effectively alleviated *Malassezia*-induced psoriatic lesions and repaired skin barrier integrity. Furthermore, the results demonstrate that OMT significantly reduced the levels of malonaldehyde, interleukin (IL)-6, IL-17, IL-23, and tumour necrosis factor (TNF)-α while promoting the activation of superoxide dismutase, catalase, and glutathione. OMT also reversed *Malassezia*-associated apoptosis and decreased the expression of the STAT3/Nf-κB/p-Nf-κB signalling pathway. Additionally, OMT reduces the nuclear expression of AhR/Nrf2 in *Malassezia*-stimulated HaCaT cells. In summary, this study demonstrated that OMT inhibits *Malassezia* biofilm formation and ameliorates *Malassezia*-associated psoriasis by modulating oxidative stress, inflammation, and apoptosis via STAT3/Nf-κB and AhR/Nrf2 pathways.

## GRAPHICAL ABSTRACT

**Figure:**
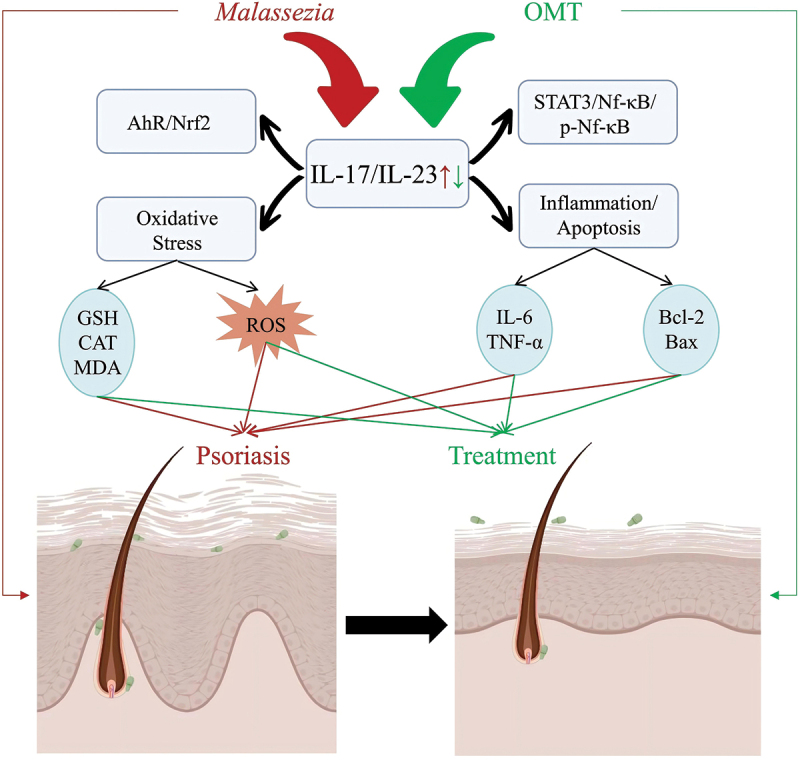

## 1. Introduction

Psoriasis is a multigenic and environmental skin disease that leads to high keratinocyte proliferation, abnormal infiltration of immune cells, and inflammation (Krane et al. 1991). As an immune-mediated inflammatory skin disease, psoriasis has been associated with the composition of the host microbiota by culture-dependent investigations and rRNA community profiling. Previous research found that psoriasis is induced or exacerbated by certain fungi, such as *Malassezia* spp. and *Candida albicans* (de Aguiar Cordeiro et al. 2023). Currently, *M. globosa* and *M. restricta* are detected in almost all psoriasis lesions patients, while other species are present in less than 30% of lesion skin (Prohic 2003). Lober et al. (1982) found that psoriasis patients developed psoriatic lesions at sites of the skin where a suspension of *Malassezia* was applied. Excessive *Malassezia* yeast colonisation in lesional skin has been found to cause psoriasis exacerbations.

Although the exact pathogenesis of psoriasis remains unclear, immune studies have highlighted the pivotal roles of inflammatory cytokines such as interleukin (IL)-17, IL-23, and tumour-necrosis factor-α (TNF-α) (Daniele et al. 2024; Tsiogkas et al. 2024). The development of psoriasis is accompanied by a nuclear factor kappa-B (Nf-κB) mediated inflammatory response. The NF-κB signalling pathway, which plays a pivotal role in regulating diverse cellular processes such as proliferation and inflammatory responses, is significantly activated in psoriatic lesions and contributes substantially to psoriasis pathogenesis (Li et al. 2012; Ma et al. 2018). In addition, there has been increasing evidence that the nuclear factor-erythroid 2-related factor (Nrf2) and the Nf-κB signalling pathways are associated with psoriasis (Bao et al. 2018; Odetayo et al. 2023). Nrf2 is a key regulator for protecting against reactive oxygen species (ROS), and it plays an important role in oxidative stress damage (Kiser et al. 2024). Oxidative stress is an essential factor that induces and aggravates psoriasis and affects the formation of a damaged stratum corneum (Cozma et al. 2023). Thus, suppressing both inflammatory mediators and anti-oxidant defence enzymes could be feasible to attenuate psoriasis.

Microorganisms have been suggested to represent external triggers that release inflammatory factors to aggravate the pathogenicity process of psoriasis (Noah 1990; Rosenberg et al. 1994). *Malassezia* is thought to induce the maturation of dendritic cells accompanied by further aggregation of inflammasomes, and the stimulation of a great number of inflammatory pathways and secretion of different cytokines with disruption of the skin barrier (Vlachos et al. 2012). Fang et al. (2022) confirmed that the abundance of *Malassezia* in lesion skin enhances the progression of psoriasis through the aberrant activation of the IL-23/IL-17 axis. This evidence suggests that *Malassezia* overgrowth in psoriatic skin can activate the inflammatory-related pathways leading to aggravation of psoriasis.

Oxymatrine (OMT), found in tangerine peels of *Sophora flavescens* (*Sophora*, Fabaceae), is an ingredient of Chinese herbal medicine. It has been shown to have anti-inflammatory and antioxidant properties (Li et al. 2011; Zhang et al. 2023). Zhou et al. (2017) observed that administration of OMT significantly improved skin lesions in patients with severe plaque psoriasis. However, detailed studies investigating the role of OMT in *Malassezia*-involved psoriasis are lacking, and the mechanism of a possible inhibitory effect of OMT on *Malassezia* yeasts remains unclear. Therefore, the present study investigated the effects of OMT on *Malassezia*-involved psoriasis in an imiquimod (IMQ)-induced mouse model and *Malassezia*-stimulated HaCaT cells.

## 2. Materials and methods

### 2.1. Reagents

OMT (C_15_H_24_N_2_O_2_, CAS: 16837-52-8, Lot No. S31408, HPLC ≥ 98%) was obtained from Shanghai Yuanye Bio-Technology Co., Ltd. (Shanghai, China). IMQ cream containing 5% imiquimod was obtained from Sichuan Med-Shine Pharmaceutical Co., Ltd. (Sichuan, China). Radioimmunoprecipitation (RIPA) buffer, polyvinylidine difluoride membranes (PVDF), NE-PER nuclear and cytoplasmic extraction kit, and bicinchoninic acid (BCA) protein assay kits were purchased from Beijing Solarbio Science & Technology Co., Ltd. (Beijing, China). Lactate dehydrogenase (LDH), triglyceride (TG), nonesterified-fatty-acids (NEFA), malonaldehyde (MDA), catalase (CAT), superoxide dismutase (SOD), and glutathione (GSH) Kits were obtained from Nanjing Jiancheng Biological Engineering Research Institute (Nanjing, China). Mouse-specific TNF-α, IL-17, IL-23, and IL-6 ELISA kits were obtained from Mskbio Co., Ltd. (Wuhan, China). Antibodies against NF-κB, STAT3, phospho-nuclear factor kappa-B (p-NF-κB), Nrf2, Aryl hydrocarbon receptor (AhR), β-actin, and horseradish peroxidase (HRP)-conjugated secondary antibody enhanced chemiluminescent detection reagents substrate were purchased from Aibotech Biotechnology Co., Ltd. (Wuhan, China). Additional chemicals and reagents, unless noted, were purchased from Sigma (St. Louis, MO, USA).

### 2.2. Microorganisms and culture media

*Malassezia globosa* (strain XH19), *M. restricta* (strain fm503), and *M. furfur* (strain CBS 1817) were grown under static conditions for 2–3 d at 37 °C in modified Dixon media (mDixon) containing malt extract (3.6%), glycerol (0.2%), peptone (0.6%), tween-40 (1%), desiccated Ox-bile (2%), and oleic acid (0.2%). For *in vivo* experiments, *Malassezia* cells were cultured for 48 to 96 h at 37 °C in liquid mDixon medium, then the medium was transferred into a 1.5 mL microcentrifuge tube and washed 3 times with phosphate-buffered salt solution (PBS) and resuspended with olive oil at a concentration of 1 × 10^9^ CFU/mL.

### 2.3. Antifungal susceptibility tests

The minimum inhibitory concentration (MIC) of *Malassezia* (1 × 10^6^ CFU/mL) to OMT was assessed using a modified CLSI M27-A2 method, using the Sabouraud dextrose broth with 1% of tween-80 (SD-T80) as previously reported (Figueredo et al. 2013). The concentration of OMT ranged from 0.4–5.12 mg/mL. Inoculated trays were incubated at 37 °C and read after 48 h. For OMT, the MIC was defined as the lowest concentration that produced a significant decrease in turbidity (≥50%) compared to the drug-free control. The minimum fungicidal concentration (MFC) of *Malassezia* strains (1 × 10^6^ CFU/mL) was analysed following the addition of different concentrations of OMT and AmB to the aqueous solutions, after which 5 μL aliquots were removed and plated without dilution on mDixon plates. After 96 h of incubation at 37 °C, a reading was made to evaluate the MFC, based on the controls’ growth. The MFC was defined as the minimal product dose required to achieve ≥ 99.9% fungicidal efficacy against *Malassezia*, demonstrated by ≤ 3 CFU/mL.

### 2.4. Till kill curves

OMT was tested at different concentrations for each test isolate. *Malassezia* cell suspensions were standardised to 1 × 10^6^ CFU/mL and inoculated into growth medium alone (control) or SD-T80 medium containing specified concentrations of OMT. At predetermined time points (2, 4, 8, 12, and 24 h), 100 μL samples were collected from each treatment group, subjected to serial dilution in sterile PBS, and 30 μL aliquots were spread onto mDixon agar plates. Following incubation at 37 °C for 72–96 h, colony counts were enumerated to determine CFU counts.

### 2.5. *Minimal biofilm inhibition concentration of OMT against* Malassezia

The inhibitory effect of OMT on biofilm formation was assessed by determining the minimal biofilm inhibition concentration (MBIC) using 96-well polystyrene plates. *M. globosa*, *M. restricta*, and *M. furfur* strains were cultured in 96-well plates with the different concentrations of OMT (0.5–5.12 mg/mL) at 37 °C for 48 h. After that, each well was washed twice with PBS, and 200 µL of 0.1% crystal violet (CV) was added into each well. The absorbance of the wells of the plate was determined at 570 nm using a microplate reader (Thermo-Scientific, Waltham, MA, USA). Biofilm inhibition rates were determined from optical density measurements and calculated using the formula (*OD*: optical density of each sample; *OD*_*A*_: optical density of growth control; *OD*_*B*_: optical density of blank control):

$$\mathrm{Inhibition}\;\mathrm{ratio}=\frac{OD-OD_{B}}{OD_{A}-OD_{B}}\times 100\%$$

### 2.6. XTT assay

For biofilm formation, *M. globosa*, *M. restricta*, and *M. furfur* strains were cultured in 96-well plates with the 1 × MIC OMT at 37 °C for 48 h. Following incubation, biofilms were gently washed with PBS to eliminate non-adherent cells, and biofilm cell metabolic activity was assessed using an XTT assay kit (Pierce et al. 2008). The absorbance in the wells of the plate was determined at 450 nm using a microplate reader. Biofilm inhibition rates were determined from optical density measurements and calculated using the formula (*OD*: optical density of each sample; *OD*_*A*_: optical density of growth control; *OD*_*B*_: optical density of blank control):

$$\text{Inhibition ratio=}\frac{OD-OD_{B}}{OD_{A}-OD_{B}}\times \text{100}\%$$

### 2.7. SYTO9/PI assay

Cell suspensions of *M. globosa*, *M. restricta*, and *M. furfur* were cultured in mDixon medium containing 1× MIC or 4× MIC of either OMT or AmB at 37 °C for 8 h. Following incubation, supernatants were carefully removed, and adherent cells on glass coverslips were washed twice with PBS. Biofilm viability was assessed by staining with SYTO9/PI (20 min, dark) and subsequent visualisation using CLSM (SYTO9: λ_ex_/_em_ 480/500 nm; PI: λ_ex_/_em_ 490/635 nm).

### 2.8. Cell culture

HaCaT keratinocytes were maintained in complete DMEM (Dulbecco’s Modified Eagle Medium) supplemented with 5% FBS, 2 mmol/L L-glutamine, and 100 U/mL penicillin-streptomycin antibiotic mixture. Cells were incubated at 37 °C in a humidified 5% CO₂ atmosphere, with medium replacement performed every 48 h. For *in vitro* experiments, *Malassezia* (1 × 10^8^ CFU/mL, mixed cultures of *M. globosa*, *M. restricta*, and *M. furfur*, the ratio is 1:1:1) were washed 3 times with cell culture media and counted. The cells were treated with *Malassezia* cells at a yeast cell to HaCaT ratio of 40:1 for 48 h.

### 2.9. Cell viability assay

To assess the cell viability of HaCaT cells, medium from the HaCaT cultures under exposure to different concentrations of OMT (1–1,000 μmol/L) were measured after 24 h by cell counting-8 (CCK-8) assay. The *OD* at 450 nm was measured using a microplate reader.

The HaCaT cells were divided into 4 groups: (1) CON (blank control); (2) *Malassezia* (HaCaT cells stimulation with mixed cultures of *M. globosa*, *M. restricta*, and *M. furfur*); (3) L-OMT (HaCaT cells stimulation with mixed cultures of *M. globosa*, *M. restricta*, and *M. furfur* and treatment with low dose OMT); (4) H-OMT (HaCaT cells stimulation with mixed cultures of *M. globosa*, *M. restricta*, and *M. furfur* and treatment with high dose OMT), and (5) AmB (HaCaT cells stimulation with mixed cultures of *M. globosa*, *M. restricta*, *M. furfur*, and treatment with AmB). The HaCaT keratinocytes in the *Malassezia* group, L-OMT, and H-OMT were infected with mixed cultures of *Malassezia* to cells at a ratio of 40:1 for 12 h. Then, the HaCaT keratinocytes in the L-OMT and H-OMT groups were treated with 100 and 300 μmol/L OMT, respectively. LDH release, as an indicator of cell membrane integrity, was measured in the supernatant from *Malassezia* and HaCaT cell co-culture supernatants using a colorimetric assay.

### 2.10. Measurement of antioxidant status

HaCaT cells (2.5 × 10^5^ cells/well) were cultured in a 6-well plate, and treatment with OMT was performed according to the above groups. The cell lysates were prepared with lysis buffer (RIPA lysis with 1% foetal calf serum, 1% foetal calf serum, and 1% PMSF) for CAT, GSH content, and MDA levels. The CAT, GSH, and MDA levels of HaCaT cells were calibrated by protein content according to the manufacturer’s instructions.

HaCaT cells (2.5 × 10^5^ cells/well) were transferred into 6-well plates and then treated with mixed cultures of *M. globosa*, *M. restricta*, and *M. furfur* and OMT as described above. ROS production in HaCaT cells was measured using DCFH₂-DA (10 μmol/L), which is oxidised to fluorescent DCF by intracellular ROS, with fluorescence quantified at 485/535 nm (λ_ex_/_em_).

### 2.11. Immunofluorescence staining

HaCaT cells (1 × 10^4^ cells/well) were stimulated with *Malassezia* at the ratio of 40:1, stimulated for 12 h, followed by being treated with OMT (100 μmol/L or 300 μmol/L for 24 h). Following OMT treatment, HaCaT cells were processed for immunofluorescence analysis. Cells were fixed and stained with primary antibodies against AhR and Nrf2, followed by FITC-conjugated secondary antibodies (λ_ex_/_em_ 488/525 nm). Nuclei were counterstained with 4’,6-diamidino-2-phenylindole (DAPI; 1 μg/mL, λ_ex_/_em_ 358/461 nm), and images were acquired using epifluorescence microscopy.

### 2.12. In vivo *experimental protocol*

BALB/c mice (6–8 weeks old) weighing 20 ± 5 g were obtained from the Beijing Vital River Laboratory Animal Technology Co., Ltd. (Beijing, China). Animals were maintained under controlled environmental conditions (22 ± 1°C, 55% ± 5% relative humidity) with a 12 h photoperiod (lights on at 07:00) and ad libitum access to standard rodent chow and filtered water. All experimental and animal handling procedures were approved by the Ethics Committee for Animal Experiments of Hebei University (HBU2025M018), and all animal experiments were conducted in accordance with the National Institutes of Health Guide for the Care and Use of Laboratory Animals (NIH Publication No. 80-23, revised 1996).

A total of 42 mice were randomly divided into (*n* = 7) the control group (CON), IMQ group (IMQ), IMQ + *Malassezia* group (IMQ + M), low-dose OMT group (L-OMT), high-dose OMT group (H-OMT), and dexamethasone group (DXM). The CON group received no OMT cream. Psoriasis-like dermatitis skin lesion was induced in all groups (except CON) by daily topical application of 5% IMQ (62.5 mg) cream on the shaved back (2 × 3 cm) for 7 consecutive days. Before applying IMQ cream, the mice in the IMQ + M group, L-OMT group, H-OMT group, and DXM group were infected with 1 × 10^8^ CFU/mL *Malassezia* (mixed cultures of *M. globosa*, *M. restricta*, and *M. furfur*) for 3 consecutive days. The mice in the L-OMT group and H-OMT group were subcutaneously injected at a dose of 50 mg/kg/day or 100 mg/kg/day, respectively, for 7 consecutive days. The mice in the DXM group were administered with DXM at a dose of 10 mg/kg/day. All animals were assessed based on the clinical psoriasis area and severity index (Li et al. 2019). The scoring ranges from 0 to 4:0, no symptoms; 1, slight symptoms; 2, moderate symptoms; 3, marked symptoms; and 4, very marked symptoms.

At the experimental endpoint (day 8), animals were sacrificed with pentobarbital (50 mg/kg i.p.). The treated dorsal skin and spleen were harvested, with skin samples either formalin-fixed for histology or snap-frozen for molecular analysis. Half of the skin tissue was homogenised with RIPA lysis (with 1% PMSF) and centrifuged at 10,000 r/min at 4 °C for 10 min. The resulting supernatant was collected for subsequent biochemical and molecular analyses.

### 2.13. Spleen index calculation

The weights of animals and spleen were collected and recorded after sacrifice. The results were expressed in g/g. The splenic index was derived by normalising spleen weight to body mass according to the equation:

SpleenIndex=spleenweight/bodyweight

### 2.14. Enzyme-linked immunosorbent assay (Elisa)

The determinations of TNF-α, IL-6, IL-23, IL-17, Bcl-2 associated X (Bax), and B-cell lymphoma-2 (Bcl-2) were accomplished by the Elisa method with skin tissue. Elisa assays were performed under the instructions of the Elisa kit.

### 2.15. Histological analysis

The skin lesions were sliced, and tissue slices were fixed in paraffin to make paraffin-embedded skin sections. Tissue sections of 4.5 μm thickness were obtained using a rotary microtome, subjected to conventional haematoxylin and eosin staining protocols, and examined under bright-field microscopy for histological assessment. For staining of oil red O in frozen skin sections (5–10 μm thick), 0.5% oil red O in propylene glycol was used according to the modified oil red O staining kit (Beyotime, Shanghai, China). For visualisation, bright-field images were captured at 400× magnification.

For immunofluorescence analysis of skin tissues, paraffin-embedded tissue sections (4.5 μm) were dewaxed with xylene and dehydrated in gradually diminishing concentrations of ethanol. Paraffin sections were stained with IL-17A (1:100 dilution; Servicebio, Cat. GB11110–1), and IL-23 (1:100 dilution; Servicebio, Cat. GB11660). The DAPI was used to stain the nucleus. Sections were observed by fluorescence microscopy (IMAGER Z2, Zeiss, Germany).

### 2.16. Western blotting

Total protein from skin lesions or HaCaT cells from each treatment group was extracted using RIPA buffer supplemented with PMSF. The analysis of the total protein of the skin tissue is described above. The protein of nuclear and cytoplasmic contents was prepared using the NE-PER nuclear and cytoplasmic extraction kit. The protein was estimated using a Bicinchoninic acid (BCA) assay kit employing BSA as a standard. Equal protein quantities (20 μg per lane) underwent electrophoretic separation (SDS-PAGE, 100 V for 90 min) and wet transfer to PVDF membranes (100 mA, 2 h). Non-specific binding sites were saturated with 5% non-fat milk in TBST (1.5 h, room temperature). After primary antibody incubation, the membranes were washed with TBS-T and then incubated with the appropriate secondary antibody. After being washed with TBS-T, the membranes were incubated with a chemiluminescence substrate and transferred for the visualisation of protein signals.

### 2.17. Statistical analysis

Results are expressed as mean ± standard error of the mean (SEM). Differences among groups were evaluated by one-way analysis of variance (ANOVA) followed by Tukey’s honestly significant difference (HSD) test for multiple comparisons. A probability value of *p* < 0.05 was considered statistically significant.

## 3. Results

### 3.1. *Inhibitory effect of OMT on* M. globosa, M. restricta, *and* M. furfur

The MIC and MFC of planktonic *M. globosa*, *M. restricta*, and *M. furfur* isolates are shown in Table 1. By the microdilution method, the 1 × MIC of OMT for *M. globosa* and *M. restricta* was 0.64 mg/mL, and the 0.5 × MIC of OMT for those two species was 0.16 and 0.32 mg/mL, respectively. Compared to *M. globosa* and *M. restricta*, *M. furfur* exhibited a higher 1 × MIC and 0.5 × MIC of OMT, which were 1.28 and 0.64 mg/mL, respectively. In addition, the MFC of OMT for *M. globosa*, *M. restricta*, and *M. furfur* were 2.56, 2.56, and 5.12 mg/mL, respectively.

**Table 1.** Inhibitory effect of OMT on *Malassezia globosa*, *M. restricta*, and *M. furfur*.

	OMT (mg/mL)		
	0.5 × MIC	1 × MIC	MFC
*Malassezia globosa*	0.16	0.64	2.56
*M. restricta*	0.32	0.64	2.56
*M. furfur*	0.64	1.28	5.12

**Figure 1.**
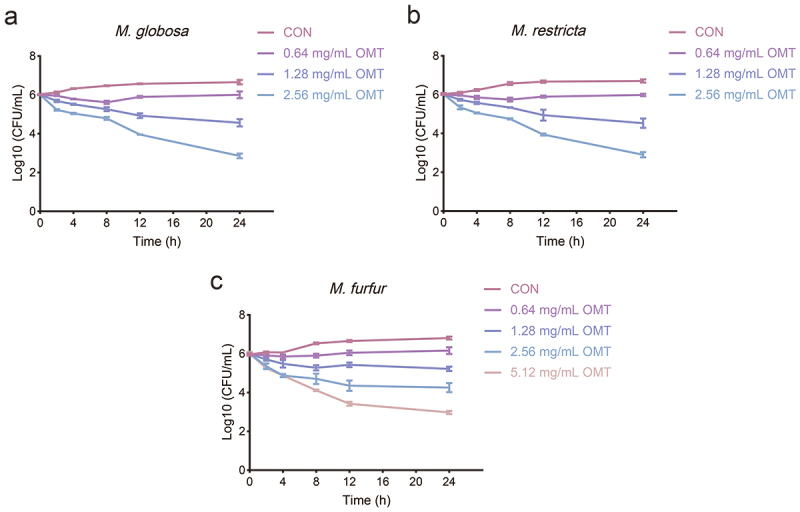
Time-kill curve for *Malassezia globosa* (a), *Malassezia restricta* (b), and *Malassezia furfur* (c) when exposed to various concentrations of OMT, respectively.

The time-killing kinetics of *M. globosa*, *M. restricta*, and *M. furfur* treatment with OMT were observed at various time intervals. The fungistatic activity of OMT for *M. globosa* and *M. restricta* at 0.64 and 1.28 mg/mL. Then, OMT exhibited the fungicidal effect against *M. globosa* and *M. restricta* at concentrations of 2.56 mg/mL OMT (Figure 1a,b). However, OMT showed fungistatic activity for *M. furfur* under 2.56 mg/mL, it exhibited the fungicidal effect against *M. furfur* was 5.12 mg/mL (Figure 1c).

### 3.2. *Inhibitory effect of OMT on biofilm formation of* M. globosa, M. restricta, *and* M. furfur

**Figure 2.**
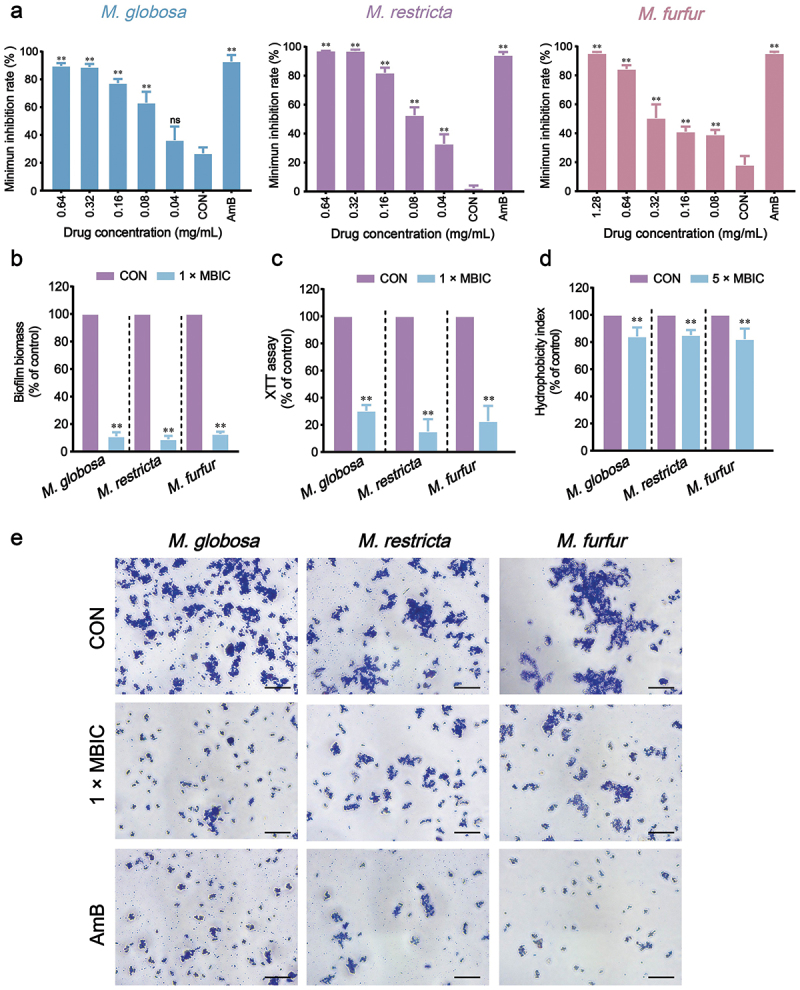
Effect of OMT on biofilm formation for *Malassezia globosa*, *Malassezia restricta*, and *Malassezia furfur*. (a) Minimal biofilm inhibition concentration (MBIC) of OMT to *M. globosa*, *M. restricta*, and *M. furfur*. (b) Biofilm biomass assay. (c) XTT assay. (d) Hydrophobicity assay. (e) Optical microscope (Magnification, 400×; scale bars = 50 µm). Values are expressed as means ± standard error of the mean (SEM), **p < 0.01 vs. CON; ns: not significant; *n* = 5.

The anti-biofilm activity of OMT against *Malassezia* (*M. globosa*, *M. restricta*, and *M. furfur*) was quantitatively assessed using the crystal violet staining assay (CVSA). The MBIC of OMT against *M. globosa*, *M. restricta*, and *M. furfur* were 0.32, 0.32, and 0.64 mg/mL, respectively (Figure 2a). Treatment with OMT at 1 × MBIC inhibited the biofilm formation of *M. globosa*, *M. restricta*, and *M. furfur* by 88.95%, 91.04%, and 87.29%, respectively, compared to the cells without OMT (Figure 2b). In addition, the concentrations at 1 × MBIC OMT inhibited the metabolic activity of *M. globosa*, *M. restricta*, and *M. furfur* compared to untreated control cells (Figure 2c). Cellular surface hydrophobicity (CSH) leads to an increased ability to form biofilms. Our results showed that the hydrophobicity was decreased after being treated with OMT at 5 × MBIC (Figure 2d). The optical microscope results also showed that OMT reduces the ability to form biofilms of *M. globosa*, *M. restricta*, and *M. furfur* (Figure 2e).

### 3.3. *Effect of OMT on the cell membrane integrity of* M. globosa, M. restricta, *and* M. furfur

**Figure 3.**
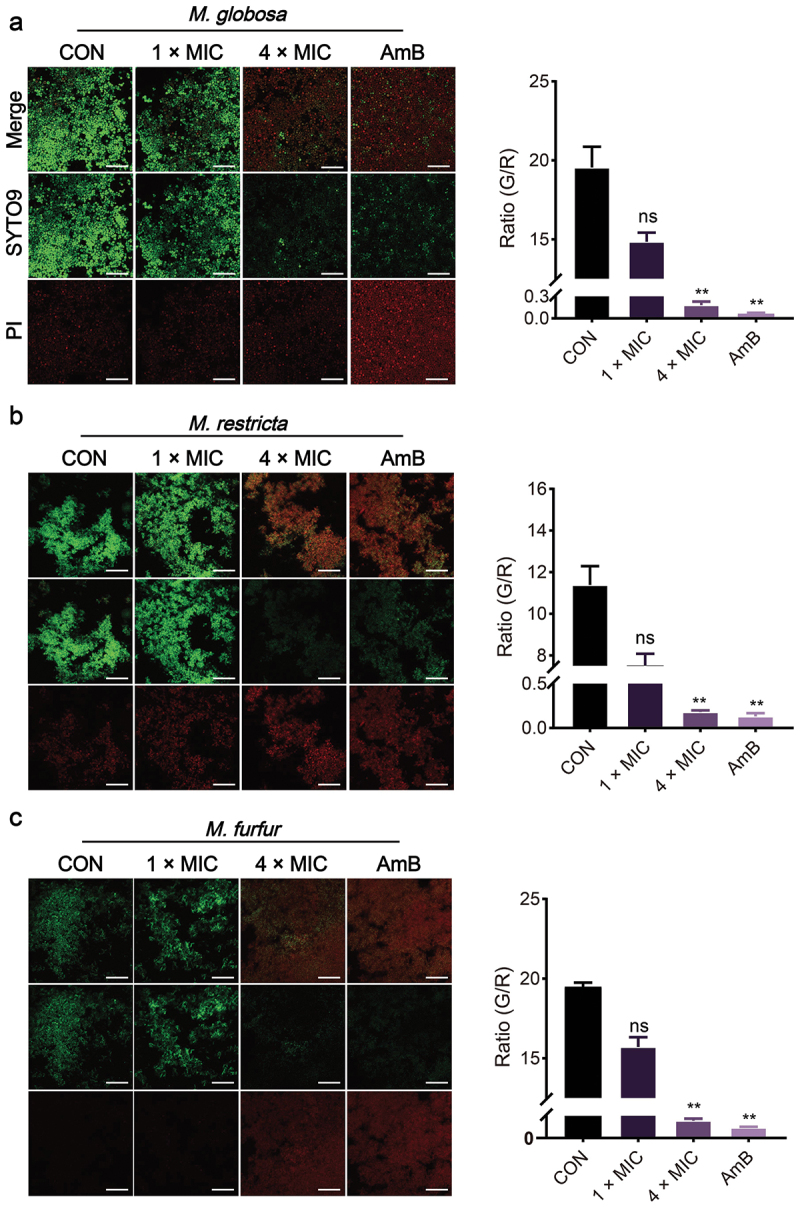
Effect of OMT against the initial cell membrane. (a) Effect of OMT on cell membrane integrity for *Malassezia globosa*. (b) Effect of OMT on cell membrane integrity for *Malassezia restricta*. (c) Effect of OMT on cell membrane integrity for *Malassezia furfur*. Magnification, 200×; scale bars = 50 µm. Values are expressed as means ± standard error of the mean (SEM), **p < 0.01 vs. CON; ns: not significant; *n* = 3.

SYTO9 can label the living fungal cells that stain green. PI can penetrate damaged membranes and stain the cells red. SYTO9/PI staining showed membrane integrity loss in *M. globosa*, *M. restricta*, and *M. furfur* following 4 × MIC OMT or AmB treatment (orange-red fluorescence), while controls maintained intact membranes (green) (Figure 3). This membrane-damaging effect correlates with OMT’s growth inhibitory capacity.

### 3.4. *Inhibitory effect of OMT on* Malassezia

**Figure 4.**
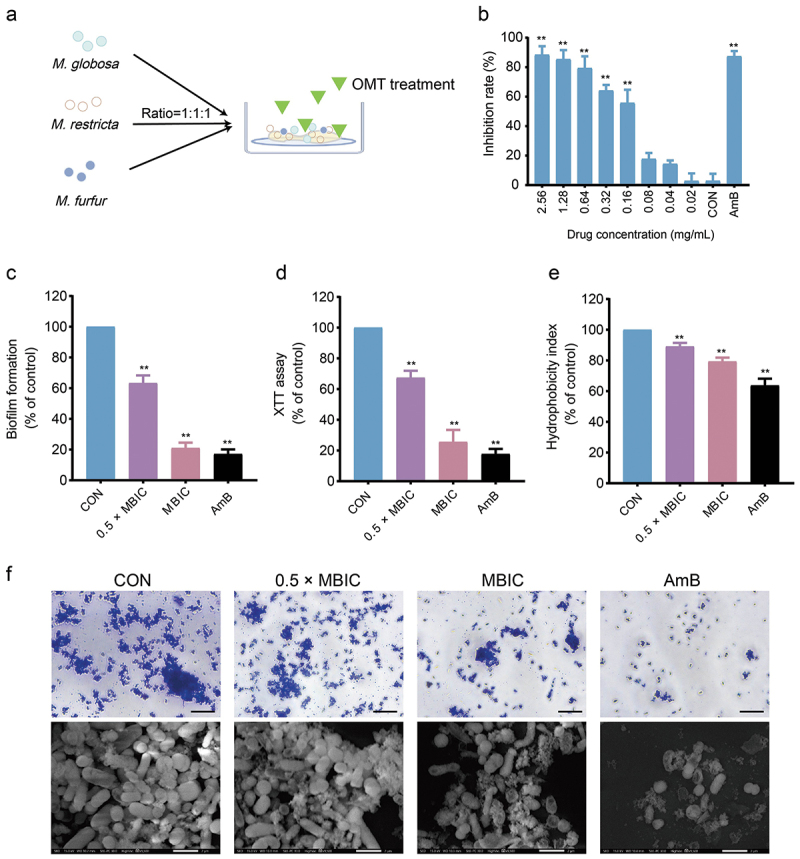
Effect of OMT on biofilm formation for *Malassezia*. (a) Scheme of abiotic surface model. (b) The MBIC of OMT to *Malassezia*. (c) Biofilm biomass assay. (d) XTT assay. (e) Hydrophobicity assay. (f) Optical microscope (Magnification, 400×; scale bars = 50 µm) and scanning electron microscope (Magnification, 9,500×; scale bars = 2 µm). Values are expressed as means ± standard error of the mean (SEM), **p < 0.01 vs. CON; *n* = 5.

To investigate the antibiofilm effect of OMT on mixed cultures of *M. globosa*, *M. restricta*, and *M. furfur* (Figure 4a). The result showed that MBIC of OMT against *Malassezia* (mixed cultures of *M. globosa*, *M. restricta*, and *M. furfur*, the ratio is 1:1:1) was 1.28 mg/mL (Figure 4b). Treatment with OMT at 1 × MBIC reduced *Malassezia*‘s biofilm biomass and metabolic activity, which means OMT can inhibit biofilm formation (Figure 4c,d). Furthermore, the hydrophobicity was decreased after treatment with OMT (Figure 4e). The optical microscope and scanning electron microscope also showed that OMT reduces the ability to form biofilms on a mixed culture of *Malassezia* (Figure 4f).

### 3.5. Effect of OMT on oxidative stress injury

**Figure 5.**
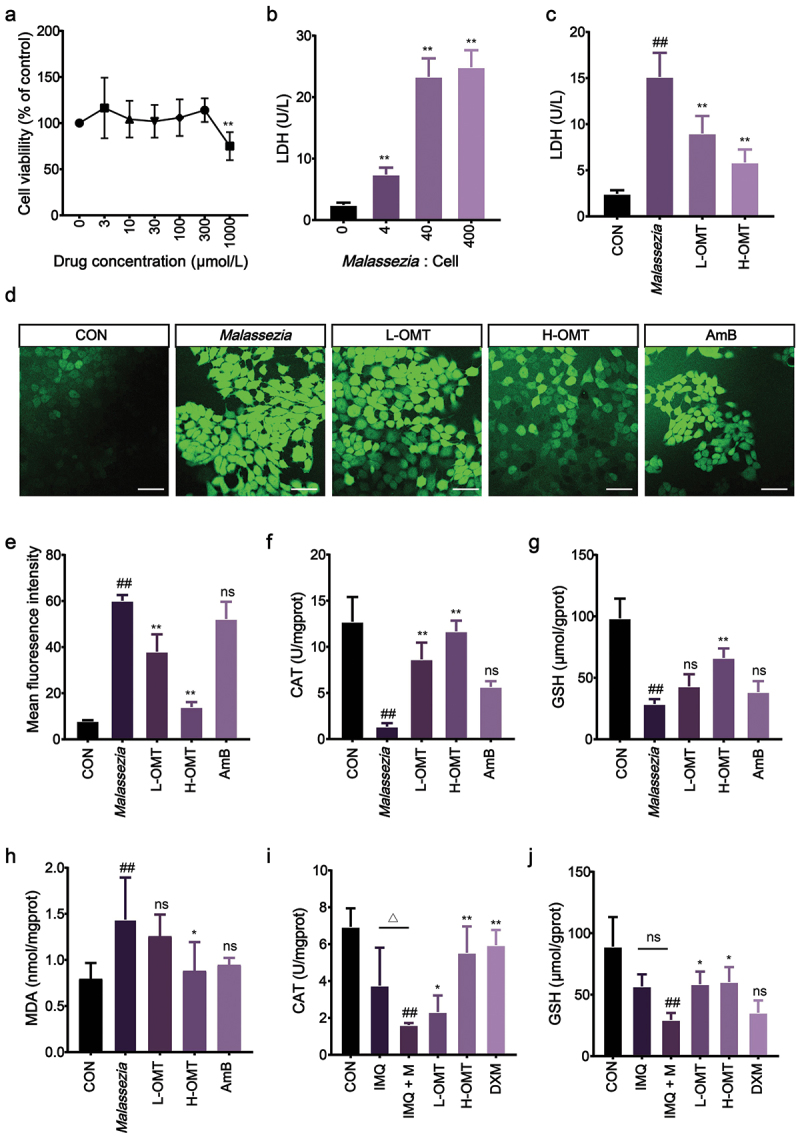
Effect of OMT on *Malassezia*-induced oxidative stress. (a) HaCaT cells were incubated with different concentrations of OMT (0–1,000 μmol/L). (b) HaCaT cell injury were infected with *Malassezia* measured by LDH assay. (c) *Malassezia*-induced HaCaT cell injury following treatment with OMT. (d, e) Representative fluorescence images showing intracellular ROS levels with DCFH2-DA (Magnification, 200×; scale bars = 50 µm). (f – h) The levels of CAT, GSH, and MDA *in vitro*. (i, j) The levels of CAT and GSH *in vivo*. CON: Control group; *Malassezia*: HaCaT cells stimulation with mixed cultures of *Malassezia globosa*, *M. restricta*, and *M. furfur*; L-OMT: HaCaT cells stimulation with mixed cultures of *M. globosa*, *M. restricta*, and *M. furfur* and treatment with low dose OMT; H-OMT: HaCaT cells stimulation with mixed cultures of *M. globosa*, *M. restricta*, and *M. furfur* and treatment with high dose OMT. Values are expressed as means ± standard error of the mean (SEM), ^##^*p* < 0.01 vs. CON; ^△^*p* < 0.05 vs. IMQ; *p < 0.05 vs. *Malassezia*; **p < 0.01 vs. *Malassezia*; ns: not significant; *n* = 5.

To determine the effect of OMT on HaCaT cells in a safe concentration, the result from CCK8 displayed that 3–300 μmol/L OMT treatment did not significantly affect the viability of HaCaT cells. For further experiments, we selected the 100 and 300 μmol/L OMT dose for use *in vitro* experiments (Figure 5a). HaCaT keratinocytes stimulated with *Malassezia* at a 40:1 ratio demonstrated significantly elevated LDH release, indicating membrane integrity compromise (Figure 5b), whereas the level of LDH was decreased in *Malassezia*-stimulated HaCaT cells after treatment with OMT (Figure 5c).

*Malassezia* infection causes oxidative stress injury, resulting in the activities of CAT and GSH reduction, but the level of MDA increases. Treatment with OMT upregulated the levels of CAT and GSH compared with the *Malassezia-*stimulated HaCaT cells (Figure 5f,g). In contrast, the content of MDA was reduced after OMT treatment (Figure 5h). Additionally, DCFH-DA was used to evaluate ROS levels in HaCaT cells. Intracellular ROS levels, measured by DCFH-DA fluorescence, showed a significant elevation in *Malassezia*-stimulated HaCaT cells compared to the CON group. OMT administration attenuated ROS generation compared with *Malassezia*-infected group (Figure 5d,e).

### 3.6. Effect of OMT on AhR/Nrf2 nuclear expression

**Figure 6.**
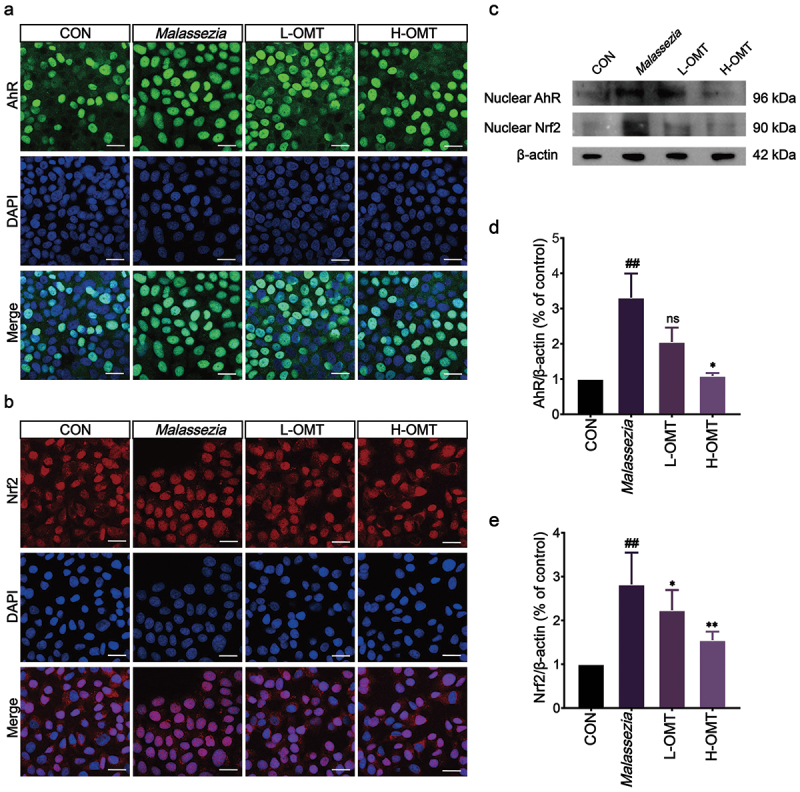
Effect of OMT on AhR expression *in vitro*. (a) Representative figures of AhR nuclear translocation in HaCaT cells (Magnification, 400×; scale bars = 25 µm). (b) Representative figures of Nrf2 nuclear translocation in HaCaT cells (Magnification, 400×; scale bars = 25 µm). (c) Representative image of bands. (d, e) The intensity of AhR and Nrf2 was standardized to protein expression levels of β-actin. CON: Control group; *Malassezia*: HaCaT cells stimulation with mixed cultures of *Malassezia globosa*, *M. restricta*, and *M. furfur*; L-OMT: HaCaT cells stimulation with mixed cultures of *M. globosa*, *M. restricta*, and *M. furfur* and treatment with low dose OMT; H-OMT: HaCaT cells stimulation with mixed cultures of *M. globosa*, *M. restricta*, and *M. furfur* and treatment with high dose OMT. Values are expressed as means ± standard error of the mean (SEM), ^##^*p* < 0.01 vs. CON; *p < 0.05 vs. *Malassezia*; **p < 0.01 vs. *Malassezia*; ns: not significant; *n* = 3.

Nuclear translocation of AhR/Nrf2 in HaCaT cells exposed to *Malassezia* after 12 h was confirmed by confocal microscopy, but treatment with OMT relieved the nuclear translocation in *Malassezia-*stimulated HaCaT cells (Figure 6a,b). Furthermore, the expression of the nuclear AhR/Nrf2 after treatment with OMT was upregulated at the protein level (Figure 6c–e).

### 3.7. Effect of OMT on psoriasis-like lesions improved

**Figure 7.**
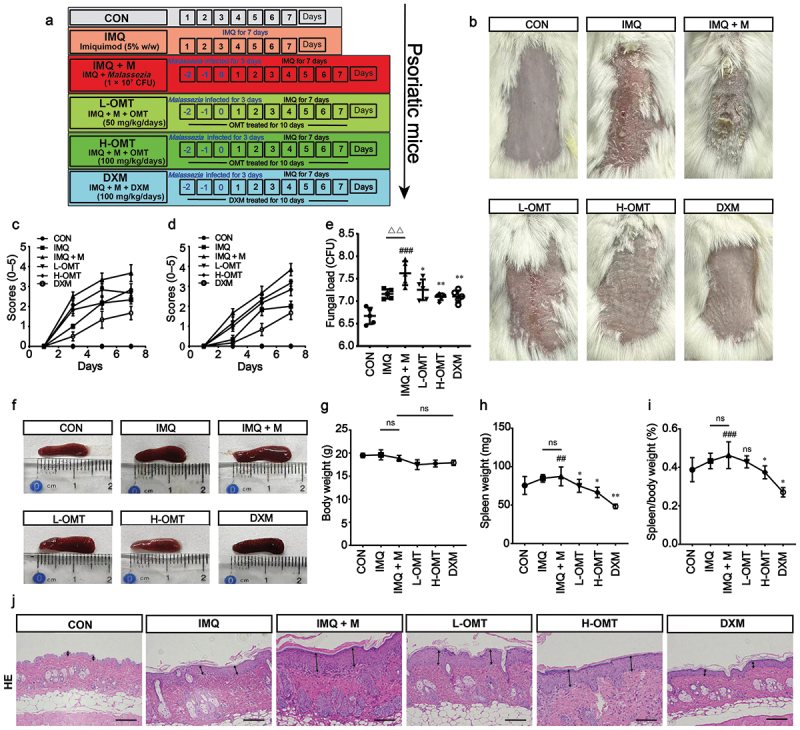
Effects of OMT on *Malassezia*-infected psoriasis-like dermatitis *in vivo*. (a) Scheme of the mice model and OMT treatments. (b) Phenotypical presentation of back skin after 7 days. (c, d) PASI score. (e) Fungal loads of skin after 7 days. (f) The photos of spleen tissue after 7 days. (g, h) Measurement of body and spleen weight. (i) Spleen index. (j) Representative HE staining results (Magnification, 200×; scale bars = 100 µm). CON: Control group; IMQ: 5% IMQ (62.5 mg) cream was application on the skin; IMQ + M: IMQ was applied to the skin after *Malassezia* infection; L-OMT: IMQ was applied to the skin after *Malassezia* infection and treatment with 50 mg/kg/day OMT; H-OMT: IMQ was applied to the skin after *Malassezia* infection and treatment with 100 mg/kg/day OMT; DXM: IMQ was applied to the skin after *Malassezia* infection and treatment with 10 mg/kg/day dexamethasone. Values are expressed as means ± standard error of the mean (SEM), ^##^*p* < 0.01 vs. CON; ^###^*p* < 0.001 vs. CON; ^△△^*p* < 0.01 vs. IMQ; *p < 0.05 vs. IMQ + M; **p < 0.01 vs. IMQ + M; ns: not significant; *n* = 5.

To investigate the therapeutic potential of OMT in *Malassezia*-exacerbated psoriasis, we established an IMQ-induced murine model exhibiting hallmark psoriatic features (Figure 7a). Subsequent *Malassezia* infection further aggravated cutaneous inflammation, mimicking clinical disease progression (Figure 7b). The PASI scores and Baker scores in the *Malassezia* and IMQ groups continuously increased after 3, 5, and 7 d. However, compared with the IMQ + M and IMQ groups, the application of OMT ameliorated the skin condition (Figure 7c–e). Histomorphometric measurements revealed that the mice infected with *Malassezia* had thicker epidermal layers than those of the IMQ group. Epidermal thickness was decreased in the L-OMT and H-OMT groups as compared with the IMQ + M group (Figure 7j).

### 3.8. Effect of OMT on spleen index

Systemic immunomodulation by OMT was assessed through splenic index quantification in our *Malassezia* associated psoriasis model. Significant enlargement of spleen size was observed in the IMQ + M group, which was subsequently attenuated by OMT treatment (Figure 7f). Fungal infection induced significant splenomegaly, which OMT treatment (50/100 mg/kg) dose-dependently attenuated the splenic index (Figure 7g–i), indicating OMT has a systemic anti-inflammatory effect.

### 3.9. Effect of OMT on skin lipid content

**Figure 8.**
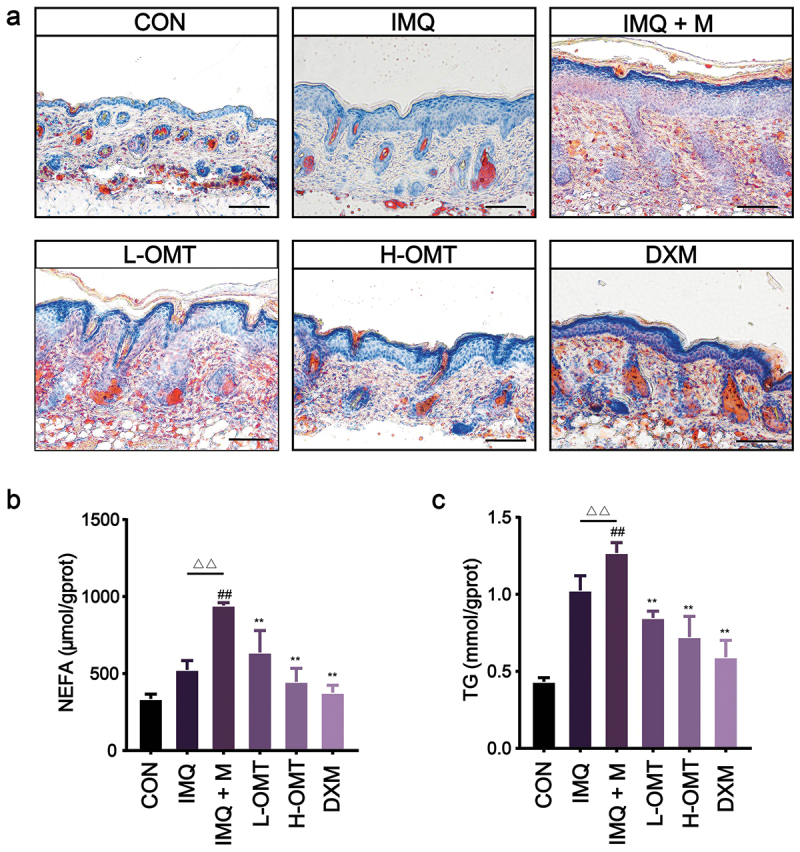
Effects of OMT on *Malassezia*-infected lipid content disorder *in vivo*. (a) Oil red O staining for skin tissue (Magnification, 200×; scale bars = 100 µm). (b, c) The levels of NEFA and TG in skin tissue. CON: Control group; IMQ: 5% IMQ (62.5 mg) cream was application on the skin; IMQ + M: IMQ was applied to the skin after *Malassezia* infection; L-OMT: IMQ was applied to the skin after *Malassezia* infection and treatment with 50 mg/kg/day OMT; H-OMT: IMQ was applied to the skin after *Malassezia* infection and treatment with 100 mg/kg/day OMT; DXM: IMQ was applied to the skin after *Malassezia* infection and treatment with 10 mg/kg/day dexamethasone. Values are expressed as means ± standard error of the mean (SEM), ^##^*p* < 0.01 vs. CON; ^△△^*p* < 0.01 vs. IMQ; **p < 0.01 vs. IMQ + M; *n* = 5.

Oil red O staining revealed that sebum accumulation in the skin cuticle increased after infection by *Malassezia*. There was no obvious lipid droplet formation in the CON and IMQ groups. However, *Malassezia*-enhanced formation of lipid droplets was decreased in the OMT-treated skin tissue (Figure 8a). The content of NEFA and TG increased in psoriasis mice infected by *Malassezia*, whereas this biomarker decreased after treatment with OMT (Figure 8b,c). This result may be due to the anti-*Malassezia* effect of OMT, which alleviates the production of lipid droplets.

### 3.10. Effect of OMT on inflammatory response

**Figure 9.**
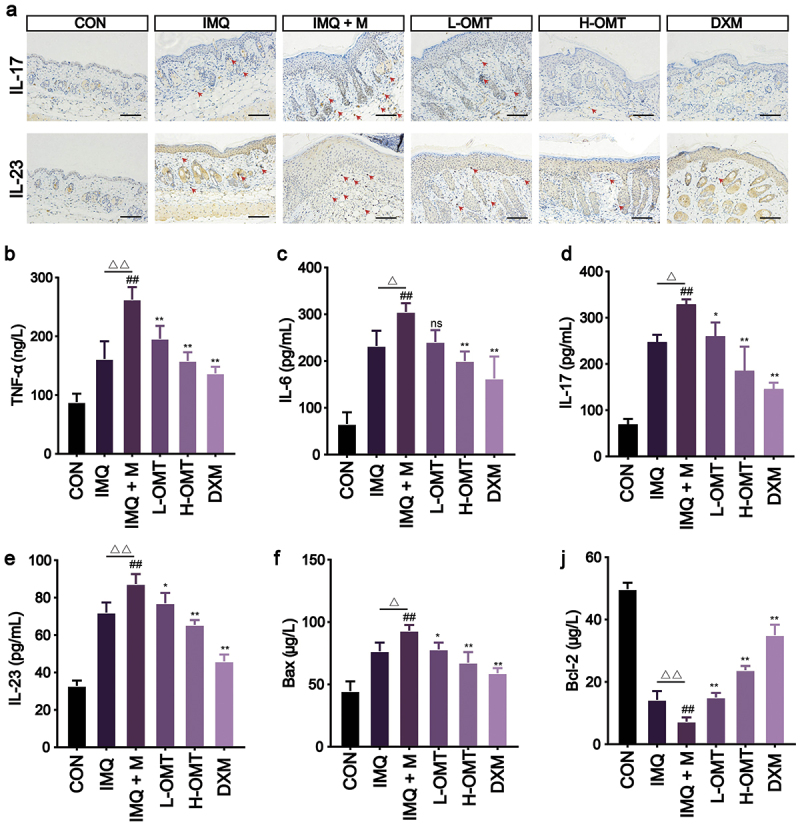
Effects of OMT on *Malassezia*-infected inflammatory factor and apoptosis expression *in vivo*. (a) Immunohistochemical staining for IL-17 and IL-23 (Magnification, 200×; scale bars = 100 µm). (b – e) The contents of TNF-α, IL-6, IL-17, and IL-23. (f – j) The levels of Bax and Bcl-2. CON: Control group; IMQ: 5% IMQ (62.5 mg) cream was application on the skin; IMQ + M: IMQ was applied to the skin after *Malassezia* infection; L-OMT: IMQ was applied to the skin after *Malassezia* infection and treatment with 50 mg/kg/day OMT; H-OMT: IMQ was applied to the skin after *Malassezia* infection and treatment with 100 mg/kg/day OMT; DXM: IMQ was applied to the skin after *Malassezia* infection and treatment with 10 mg/kg/day dexamethasone. Values are expressed as means ± standard error of the mean (SEM), ^##^*p* < 0.01 vs. CON; ^△^*p* < 0.05 vs. IMQ; ^△△^*p* < 0.01 vs. IMQ; *p < 0.05 vs. IMQ + M; **p < 0.01 vs. IMQ + M; ns: not significant; *n* = 5.

Immunohistochemical staining was used to detect the expression of IL-17 and IL-23, and dark brown stained tissue represents a positive staining. The expression of IL-17 and IL-23 was upregulated after infection with *Malassezia* compared with the IMQ and CON groups. However, the cytokine levels of IL-17 and IL-23 can be reversed after OMT treatment (Figure 9a).

ELISA quantification revealed that *Malassezia* infection elevated key psoriatic cytokines (IL-6, IL-17, IL-23, and TNF-α) compared to the IMQ alone group. However, OMT and DXM treatment reduced these proinflammatory cytokine expressions (Figure 9b–f). Persistent inflammation results in cell apoptosis. Thus, the level of Bax was found to be decreased and that of Bcl-2 increased in the *Malassezia* group, and after treatment with OMT, the content of Bax was upregulated and that of Bcl-2 reduced (Figure 9g,h).

### 3.11. Effect of OMT on STAT3/Nf-κB/p-Nf-κB

**Figure 10.**
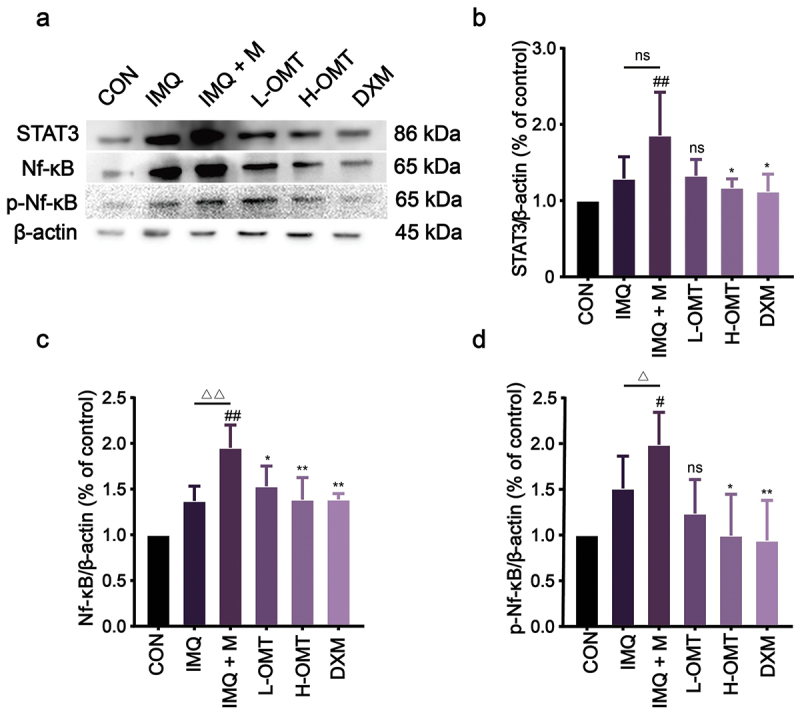
Effects of OMT on STAT3/Nf-κB pathways in dorsal skin *in vivo*. (a) Representative image of bands. (b – d) The intensity of STAT3, Nf-κB, and p-Nf-κB was standardized to protein expression levels of β-actin. CON: Control group; IMQ: 5% IMQ (62.5 mg) cream was application on the skin; IMQ + M: IMQ was applied to the skin after *Malassezia* infection; L-OMT: IMQ was applied to the skin after *Malassezia* infection and treatment with 50 mg/kg/day OMT; H-OMT: IMQ was applied to the skin after *Malassezia* infection and treatment with 100 mg/kg/day OMT; DXM: IMQ was applied to the skin after *Malassezia* infection and treatment with 10 mg/kg/day dexamethasone. Values are expressed as means ± standard error of the mean (SEM), ^#^*p* < 0.05 vs. CON; ^##^*p* < 0.01 vs. CON; ^△^*p* < 0.05 vs. IMQ; ^△△^*p* < 0.01 vs. IMQ; *p < 0.05 vs. IMQ + M; **p < 0.01 vs. IMQ + M; ns: not significant; n = 3.

The anti-inflammatory effect of OMT was confirmed by immunoblot analysis of STAT3/Nf-κB/p-Nf-κB proteins to identify the factors. Western blot analysis revealed marked upregulation of STAT3/Nf-κB/p-Nf-κB in *Malassezia*-infected skin, while OMT treatment attenuated these signalling cascades (Figure 10).

## 4. Discussion

Psoriasis, a chronic inflammatory skin disorder affecting approximately 2% of the global population, clinically manifests as erythematous, scaly plaques with epidermal thickening. The role of *Malassezia* spp. in psoriasis is still unclear, but several reports have associated these yeasts with the development of skin lesions in psoriasis (Baroni et al. 2004; Zomorodian et al. 2008). Amaya et al. (2007) detected *M. globosa* and *M. restricta* at high rates in the skin of psoriasis patients. OMT is a natural bioactive compound extracted from *Sophora flavescens* with anti‑inflammatory, antioxidant, antiviral, and antiproliferative properties (Yuan et al. 2011). A clinical trial showed that OMT effectively improved psoriatic skin lesions by inhibiting the proliferation of epidermal cells. More importantly, the adverse effects of OMT were less and milder. Furthermore, the antifungal activity of OMT has been reported, and it may have an antifungal effect on *Aspergillus fumigatus* by destroying the cell wall and cell membrane (Liu et al. 2024). Nevertheless, the antifungal and antibiofilm effects of OMT on *Malassezia*-involved psoriasis are unclear. This study explored the antifungal effect of OMT on *Malassezia* by assessing biofilm cell quantification using a CVSA assay, an XTT reduction assay, and microscopic analyses of biofilm formation. In addition, this work elucidated the mechanism of OMT in alleviating *Malassezia*-involved psoriasis and confirmed the protective effect on psoriasis-like lesions using a *Malassezia*-infected mouse model.

The antimicrobial activity evaluation revealed that OMT against *Malassezia* and the MIC of OMT on the planktonic cells of *M. globosa*, *M. restricta*, and *M. furfur* were 0.64, 0.64, and 1.28 mg/mL, respectively, which means that OMT has an inhibitory effect on *Malassezia* yeasts (Table 1). Fungistatic drugs are defined as those that inhibit fungal growth, whereas fungicidal drugs essentially kill all (>99.9%) cells in a fungal population. For *M. globosa* and *M. restricta*, the time-kill assays exhibited the fungicidal effect of OMT at concentrations of 8 × MIC. In contrast, only the fungistatic effect of OMT at 8 × MIC for *M. furfur* (Figure 1). *Malassezia*-based biofilms tightly attach to the skin (Li et al. 2018). A common characteristic of biofilms, compared with their free-floating counterparts, is the greater resistance of their cells to drugs (Zhou et al. 2024). In addition, the formation of biofilm leads to the chronic status of many diseases caused by microorganisms. *In vitro* biofilm formation has correlated well with *in vivo* and *ex vivo* biofilm models. The MBIC of OMT on *M. globosa*, *M. restricta*, and *M. furfur* were 1.28, 1.28, and 2.56 mg/mL, respectively (Figure 2a,b). Then, we investigated the effect of OMT on biofilm formation on abiotic surfaces using the XTT assay. The results obtained showed that OMT inhibited the biofilm formation of *Malassezia* at 1 × MIC, and the amount of purple patches in the untreated biofilms was decreased compared to the OMT treatment group (Figure 2c,e). Hydrophobicity and biofilm development are closely linked in various fungi and, therefore, the former can be useful in predicting the propensity of different strains to form biofilm (Habimana et al. 2011). Thus, the CSH of *M. globosa*, *M. restricta*, and *M. furfur* reduced by OMT at 5 × MIC was partially correlated with the antibiofilm efficacies (Figure 2d). Meanwhile, the result of the visualisation of cell membrane integrity showed that OMT at a MIC damage the cell membrane integrity of *Malassezia*, as evidenced by a similar increase in bright red fluorescence (Figure 3). Thus, the anti-*Malassezia* mechanisms of OMT may involve antibiofilm formation and disrupting cell membranes.

AhR is a cytosolic transcription factor with multiple functions in skin physiology, such as participation in wound healing, stress response to ultraviolet B, melanogenesis, and inflammation (Hidaka et al. 2019; Aoki and Orfali 2023). *Malassezia* species produce AhR ligands when L-tryptophan is used as the nitrogen source in the sweat (Kramer et al. 2005). HaCaT keratinocytes infected with *Malassezia pachydermatis* exhibited significant AhR nuclear translocation and subsequent upregulation of CYP1A1/CYP1B1 mRNA expression (Buommino et al. 2018). Herein, the expression of AhR was enhanced after stimulation with *Malassezia* and this result was consistent with the previously reported. Treatment with OMT in HaCaT cells efficiently reduced the AhR levels (Figure 6a). AhR nuclear translocation induces an anti-oxidant response through the activation of the nuclear factor-erythroid factor 2-related factor 2 (Nrf2) (Fusco et al. 2023). As data showed, *Malassezia* stimulated the HaCaT cells and activated Nrf2 expression, but it can be reduced after treatment with OMT (Figure 6b). Nrf2 acts as a key regulator for protecting against ROS, and it plays an important role in oxidative stress (Qi et al. 2024). Then, the activation of the Nrf2 pathway in psoriasis creates a cascading effect that results in the production of several powerful antioxidants, including glutathione (GSH) and catalase (CAT) (Xing et al. 2024). We found that the levels of ROS, GSH, and CAT were decreased after OMT treatment, which means OMT has an antioxidant capacity to protect HaCaT cells from oxidative damage caused by *Malassezia* stimulation (Figure 5).

Our result confirmed that the abundance of *Malassezia* expression is positively correlated with the PASI score and increased *Malassezia* cell densities significantly exacerbated psoriasis in mice (Figure 7b). This phenomenon is consistent with clinical results obtained by Fang et al. (2022). Thus, we further evaluated the effects of OMT on psoriasis caused by *Malassezia* overgrowth. The results showed that OMT could effectively reduce erythema and scaling of the skin and inhibit cuticle thickening (Figure 7c–e). In addition, the spleen size of the mice was also restored from enlarged to near normal after OMT treatment (Figure 7g–j). Furthermore, intercellular lipid content is a component of the skin barrier. We can see that *Malassezia*-mediated skin barrier disruption aggravates epidermal intercellular lipids in psoriatic mice skin but the lipid content is restored with OMT treatment (Figure 8a). Various lipolytic enzymes, including lipase, produced by *Malassezia* species help in the utilisation of essential fatty acids from exogenous lipid sources (Triana et al. 2017). Thus, the concentration of NEFA was measured by a colorimetric method. The content of NEFA was found to be increased in the *Malassezia*-infected group, but it reduced after treatment with OMT. In addition, TG from the sebaceous glands is the nutrient source of *Malassezia*, and OMT-decreased TG production is likely to reduce *Malassezia* proliferation indirectly (Figure 8b,c).

Current knowledge suggests that the overexpression of IL-17 and IL-23 is of pathologic significance in psoriatic initiation, maintenance, and recurrence (Quah et al. 2024). It was shown that colonisation of the skin by *Malassezia* yeasts can strengthen inflammation via the IL-17/IL-23 axis. The cytokine levels, including IL-6, IL-17, IL-23, and TNF-α, increased in the *Malassezia* overexpression group. However, IL-6, IL-17, IL-23, and TNF-α showed a decrease after treatment with OMT compared with the IMQ and IMQ + M group (Figure 9a–f). Previous studies have hinted that the above-mentioned inflammatory cytokines are regulated via STAT3 and Nf-κB signalling pathways, which are two major inflammatory pathways (Huang et al. 2024). The functional interaction between Nf-κB and STAT3 transcriptional activities for maximum induction and activation of cytokines, such as IL-6 and TNF-α (Zhou et al. 2023). In comparison with healthy mice, the levels of Nf-κB and STAT3 were significantly higher in *Malassezia*-mediated psoriasis (Figure 10). Activation of Nf-κB induces the release of several pro-inflammatory cytokines and leads to apoptosis. Notably, apoptosis was inhibited after treatment with OMT, which was evidenced by the significant increase in the Bax/Bcl-2 ratio, an indicator of the initiation of apoptosis (Figure 9f–j). These results support that OMT has a multifactorial protective effect against *Malassezia*-mediated psoriasis.

Taken together, our data investigated the antifungal effect of OMT on *Malassezia* through the inhibition of biofilm formation. Additionally, OMT may protect HaCaT cells against *Malassezia*-induced damage by exerting antioxidant effects and modulating the AhR/Nrf2 signalling pathways. Our data show the protective effect of OMT on *Malassezia*-infected psoriasis through inhibiting inflammation and apoptosis *in vivo*. Thus, OMT demonstrates dual therapeutic effects by both treating psoriasis and inhibiting *Malassezia* overgrowth, ultimately leading to improved disease prognosis.

## Abbreviations

AhR: Aryl hydrocarbon receptor
Bax: Bcl-2 associated X protein
Bcl-2: B-cell lymphoma-2
CAT: Catalase
CCK-8: Cell counting kit-8
CVSA: Crystal violet staining assay
DXM: Dexamethasone
GSH: Glutathione
HE: Haematoxylin and eosin
IL-6: Interleukin-6
IL-17: Interleukin-17
IL-23: Interleukin-23
IMQ: Imiquimod
LDH: Lactate dehydrogenase
MDA: Malondialdehyde
MBIC: Minimal biofilm inhibition concentration
MFC: Minimum fungicidal concentration
MIC: Minimum inhibitory concentrations
NEFA: Nonesterified-fatty-acids
Nf-κB: Nuclear factor Κb
Nrf2: Nuclear factor-erythroid 2-related factor
OMT: Oxymatrine
PASI: Psoriasis area severity index
ROS: Reactive oxygen species
SOD: Superoxide dismutase
STAT: Signal transducer and activator of transcription
TG: Triglyceride
TNF-α: Tumour necrosis factor-α
